# Antibiotic activity of telithromycin and comparators against bacterial pathogens isolated from 3,043 patients with acute exacerbation of chronic bronchitis

**DOI:** 10.1186/1476-0711-4-5

**Published:** 2005-03-08

**Authors:** Sanjay Sethi, Antonio Anzueto, David J Farrell

**Affiliations:** 1 University at Buffalo SUNY, Buffalo NY, USA; 2 South Texas Veterans Health Care System, Audie L. Murphy Memorial Veterans Hospital Division, San Antonio, USA; 3 GR Micro Limited, London, UK

## Abstract

**Background:**

Antimicrobial therapy is considered an important component in the medical management of most patients with acute exacerbation of chronic bronchitis (AECB). The three predominant bacterial species isolated are nontypeable *Haemophilus influenzae*, *Moraxella catarrhalis*, and *Streptococcus pneumoniae*. *Staphylococcus aureus *is also frequently isolated while atypical bacteria are thought to cause up to 10% of exacerbations. Antibacterial resistance is increasing worldwide and little surveillance data exist concerning pathogens isolated from patients with AECB.

**Methods:**

This study examines the prevalence of antibacterial resistance in isolates obtained from patients with clinically diagnosed AECB. A total of 3043 isolates were obtained from 85 centres in 29 countries, between 1999–2003, and were tested against the new ketolide telithromycin and a panel of commonly used antibiotics.

**Results and Discussion:**

Of the *S. pneumoniae *isolates, 99.9% were susceptible to telithromycin, but only 71% were susceptible to erythromycin and 75.3% to penicillin. Of the *H. influenzae *isolates, 99.6% were susceptible to telithromycin. 11.7% of these isolates produced β-lactamase. Almost 10% of *S. pneumoniae *were multidrug-resistant; 99.0% of these isolates were susceptible to telithromycin. Telithromycin also demonstrated good *in vitro *activity against *M. catarrhalis *(MIC_90 _= 0.12 mg/L) and was the most active compound against methicillin-susceptible *S. aureus *(98.9% susceptible).

**Conclusion:**

Telithromycin demonstrated similar or better activity against the bacterial species investigated than the other agents, with the most complete coverage overall. These species are the predominant causative bacterial pathogens in AECB and thus the spectrum of activity of telithromycin makes it a potential alternative for the empirical treatment of AECB.

## Introduction

The World Health Organization (WHO) estimates that chronic obstructive pulmonary disease (COPD) is the fourth leading cause of death worldwide. In the year 2000, it was estimated that 2.74 million people died from COPD worldwide [1]. COPD is defined by the presence of irreversible or partially irreversible airway obstruction in patients with chronic bronchitis or emphysema [2,3]. The disease is characterized by recurrent (1–4 per year) acute exacerbations of chronic bronchitis (AECB), defined by a subjective increase from baseline of one or more symptoms including shortness of breath, cough, sputum production, and sputum purulence [4]. The precipitating factors for AECB have been extensively researched and determined to be heterogeneous with complex aetiology [5-10].

Results from a number of placebo-controlled clinical investigations have demonstrated that antibacterial agents are of significant clinical benefit in the treatment of AECB, particularly for those patients with at least two of the three cardinal symptoms of AECB (worsening dyspnoea, increased sputum volume, and increased sputum purulence) and/or severe airway obstruction [11-13]. Other clinical trials measuring non-traditional endpoints have shown that antibiotic therapy reduces the time to symptom resolution and has long-term benefits including greater intervals between episodes of exacerbation [14,15]. Consequently, antibiotic therapy is considered an important component in the medical management of patients with AECB.

Bacteria can be isolated from 40–60% of sputum samples of patients experiencing AECB [16]. The three predominant bacterial species isolated are non-typeable *Haemophilus influenzae*, *Moraxella catarrhalis*, and *Streptococcus pneumoniae*. Other less frequently isolated potential pathogens are Gram-negative enterobacteria, *Haemophilus parainfluenzae*, *Staphylococcus aureus*, and *Pseudomonas aeruginosa*. Gram negative enterobacteria and *Pseudomonas aeruginosa *are more frequently isolated in patients with severe underlying disease [10]. Viral infections are present in approximately 30% of exacerbations, *Mycoplasma pneumoniae *in 1–10%, and *Chlamydophila pneumoniae *in 4–5% (serologically identified) [6-10].

Amoxycillin, ampicillin, sulfamethoxazole-trimethoprim (trimethoprim-sulphamethoxazole), tetracyclines, and erythromycin are considered first-line antimicrobial therapy for AECB [17]. The clinical utility of these agents is, however, being hampered by the increasing global spread of pathogens with resistance to one or more of these agents. Up to 40% of *H. influenzae *isolates and more than 90% of *M. catarrhalis *isolates produce β-lactamase and this limits the value of penicillins and some other β-lactams [18]. Furthermore, resistance to penicillin and macrolides has spread rapidly among isolates of *S. pneumoniae *[19]. Other agents used include extended spectrum cephalosporins, amoxycillin/clavulanate, azithromycin, clarithromycin, and levofloxacin.

Telithromycin is the first ketolide available for clinical use. Derivatives of erythromycin-A, the ketolides, like the macrolides, exert their antimicrobial action by binding to the bacterial ribosome. Although both macrolides and ketolides bind strongly to a region of domain V in the 23S rRNA of the ribosome, telithromycin has additional strong binding to a region in domain II to which the macrolides bind weakly [20]. Ketolides are also poor substrates for the efflux pump (mefA) responsible for macrolide resistance in *S. pneumoniae *[21]. Consequently, telithromycin has been found to have potent activity against macrolide resistant *S. pneumoniae *with methylase, efflux or ribosomal mutations as the mechanisms of resistance [22,23].

There is a need for alterative therapeutic options for the treatment of AECB and surveillance data are needed to help determine the suitability of new agents. The PROTEKT (Prospective Resistant Organism Tracking and Epidemiology for the Ketolide Telithromycin) study is an international, longitudinal, antibacterial resistance surveillance study, which was initiated in 1999 to monitor the spread of resistance among respiratory tract pathogens worldwide. Here we analyze the *in vitro *antimicrobial activity of bacterial isolates obtained from patients clinically diagnosed with AECB in 3 consecutive years of the PROTEKT study. Using these data, and previously published clinical data, the potential role of telithromycin in the treatment of AECB will be discussed.

## Materials and Methods

### Patients and bacterial isolates

Details of the study design, including the selection of patients and the methodology for the identification of isolates and their storage in the PROTEKT study has been described previously [24]. Isolates in this study were obtained from patients diagnosed with AECB from in 85 centres in 29 countries (Table 1). To be included in this analysis, an isolate was deemed pathogenic in AECB by clinical and laboratory findings. Isolates were only acceptable if the patient was ≥ 30 years old and the specimen was obtained from blood, bronchoalveolar lavage (BAL), or sputum. Isolates from patients diagnosed with AECB obtained from other sites (e.g., ear, throat, nasopharynx) and isolates obtained from patients <30 years of age were excluded from this analysis because AECB is more likely to be present in patients ≥ 30 years of age and the responsible bacterial pathogen is more likely to be correctly isolated from the blood, BAL, or sputum.

**Table 1:** Geographical distribution of isolates from AECB patients used in this study

Area	Countries	Centres	Isolates
North America	2	4	319
South America	6	14	427
Europe	13	41	1847
Australasia	6	19	437
South Africa	1	6	13

Totals	29	85	3043

In Year 1 (1999–2000), each centre had a quota of 60 isolates of *S. pneumoniae*, 40 *H. influenzae*, 15 *H. parainfluenzae*, 20 *M. catarrhalis*, 25 *Streptococcus pyogenes *and 20 *S. aureus *to collect. In years 2 (2000–2001) and 3 (2001–2002), *H. parainfluenzae *were not collected and 15 extra isolates of *S. pneumoniae *were collected instead.

### Antimicrobial testing

The comparator agents used were four β-lactams; penicillin (for *S. pneumoniae *and *S. aureus*), ampicillin (for *H. influenzae*, *H. parainfluenzae *and *M. catarrhalis*), amoxycillin/clavulanate, and cefuroxime, three macrolides/azalides; erythromycin, clarithromycin, and azithromycin, the folate synthesis inhibitor; trimethoprim-sulphamethoxazole, the tetracycline; tetracycline and a fluoroquinolone, levofloxacin.

Minimum inhibitory concentrations (MIC) of each antibacterial were determined using the National Committee for Clinical and Laboratory Standards (NCCLS) broth microdilution methodology and lyophilised microtitre plates (Sensititre, Trek Diagnostics) at a central laboratory (GR Micro Ltd., London, UK) [26]. NCCLS breakpoints [25,26] were used to interpret the MIC data and to determine susceptibility status. The NCCLS breakpoints for telithromycin for *S. pneumoniae *and for *S. aureus *are ≤ 1 mg/l is susceptible, 2 mg/l is intermediate, and ≥ 4 mg/l is resistant, and for *H. influenzae *≤ 4 mg/l is susceptible, 8 mg/l is intermediate, and ≥ 16 mg/l is resistant [27].

## Results

A total of 3043 bacterial pathogens were isolated from patients in 29 countries around the world, with by far the largest number of specimens (1841, 60.5%) coming from Europe (Table 1). Percentage of isolates by country were as follows: Argentina 8.0%, Australia 1.1%, Austria 0.6%, Brazil 4.0%, Canada 9.1%, China 1.7%, Colombia 0.1%, Ecuador 0.6%, Eire 0.03%, France 3.4%, Germany 14.3%, Hungary 1.2%, Indonesia 0.03%, Italy 18.9%, Japan 10.0%, Mexico 1.3%, Poland 10.2%, Portugal 2.8%, Russia 0.2%, South Africa 0.4%, South Korea 0.9%, Spain 5.3%, Sweden 0.4%, Switzerland 0.6%, Taiwan 0.7%, Turkey 0.3%, United Kingdom 2.4%, United States 1.4%, Venezuela 0.1%.

Of these isolates identified as causative pathogens for bacterial AECB, *S. pneumoniae *and *H. influenzae *formed the majority (1075 and 1037 respectively), followed by *M. catarrhalis *(536) (Table 2). Patients were predominantly male (63.5%), with 47.5% of patients belonging to the (30–64) year age group and 52.5% in the >64 year old age group. No difference in the distribution of pathogens by age group was observed (data not shown).

**Table 2:** Distribution of specimen types by species for the 3043 bacterial pathogens described in this study

**Specimen**	*S. pneumoniae*	*H. influenzae*	*M. catarrhalis*	*S. aureus*	*H. parainfluenzae*^1^	**Total **[n (%)]
**Sputum**	832	895	492	219	43	2481 (81.5)
**BAL^2^**	144	135	44	66	17	406 (13.4)
**Blood**	99	7	0	50	0	156 (5.1)

**Total **[n (%)]	1075 (35.3)	1037 (34.1)	536 (17.6)	335 (11.0)	60 (2.0)	3043 (100)

^1^Only isolated in the first year of the study

^2^Bronchoalveolar lavage

Table 3 shows the range of MIC values, the MIC_50 _and MIC_90 _of the various agents against the five species. Where breakpoints were available the percentage of isolates to the various agents is also included. Telithromycin had similar or better *in vitro *susceptibility than the comparator agents against all of these species. Activity against *S. pneumoniae *was particularly good, with telithromycin being the most active agent; 99.9% of isolates were classified as susceptible and the MIC_90 _(0.12 mg/L) was substantially lower than all other compounds tested.

**Table 3:** *In vitro *activity of antibacterial agents against 3043 bacterial pathogens isolated from patients with AECB and % susceptibilities to antibacterial agents.

**Organism**	**Antibacterial**	**MIC mg/l**			**% susceptible**		
		**range**	**50**	**90**	**total**	**MSSA**	**MRSA**
*S. pneumoniae *N = 1075	Telithromycin	0.004–2	0.015	0.12	99.9		
	Azithromycin	0.03->64	0.12	>64	71.2		
	Clarithromycin	0.015->32	0.03	>32	71		
	Erythromycin	0.03->64	0.06	>64	71		
	Penicillin	0.008–8	0.03	2	75.3		
	Amox/clavulanate^1^	0.015–8	0.03	2	96.1		
	Cefuroxime	0.015–16	0.03	2	82.2		
	Trimethoprim-sulphamethoxazole	0.12–32	0.25	8	62		
	Tetracycline	0.12–32	0.25	32	69.6		
	Levofloxacin	0.5->32	1	1	98.9		
							
*H. influenzae *N = 1037	Telithromycin	0.002–16	1	2	99.6		
	Azithromycin	0.06–32	1	2	99.7		
	Clarithromycin	0.25->64	8	16	82.4		
	Erythromycin	0.25->64	4	8	-^2^		
	Ampicillin	0.12–32	0.25	16	87.3		
	Amox/clavulanate^1^	0.12–4	0.5	1	100		
	Cefuroxime	0.12–16	1	2	99.5		
	Trimethoprim-sulphamethoxazole	0.03–32	0.06	4	80.7		
	Tetracycline	0.12–32	0.5	1	97.4		
	Levofloxacin	0.008–8	0.015	0.015	99.8		
							
*M. catarrhalis *N = 536	Telithromycin	0.004–0.5	0.06	0.12			
	Azithromycin	0.06–0.25	0.06	0.06			
	Clarithromycin	0.25–0.5	0.25	0.25			
	Erythromycin	0.25–1	0.25	0.25			
	Ampicillin	0.12–32	8	16			
	Amox/clavulanate^1^	0.12–0.5	0.12	0.25			
	Cefuroxime	0.12–16	1	2			
	Trimethoprim-sulphamethoxazole	0.06–4	0.25	0.5			
	Tetracycline	0.12–32	0.25	0.5			
	Levofloxacin	0.008–0.06	0.03	0.03			
							
*S. aureus *N = 335	Telithromycin	0.03->32	0.06	>32	85.1	98.9	24.2
	Azithromycin	0.12->64	1	>64	70.4	84.2	9.7
	Clarithromycin	0.03->32	0.25	>32	70.4	84.2	9.7
	Erythromycin	0.12->64	0.25	>64	70.4	84.6	9.7
	Penicillin	0.008–8	4	8	23.6	28.9	0
	Amox/clavulanate^1^	0.06–8	0.5	8	83.1	100	0
	Cefuroxime	0.12–16	1	16	81.2	100	0
	Trimethoprim-sulphamethoxazole	0.12–32	0.12	0.25	94.9	97.4	83.9
	Tetracycline	0.12–32	0.5	32	84.8	92.7	50
	Levofloxacin	0.5–64	0.5	8	76.7	81.7	6.5
							
*H. parainfluenzae *N = 60	Telithromycin	0.06–4	1	2	100		
	Azithromycin	0.06–2	0.5	1	100		
	Clarithromycin	0.25–16	4	8	93.3		
	Erythromycin	0.5–8	2	4	-^2^		
	Ampicillin	0.12–32	0.25	1	90		
	Amox/clavulanate^1^	0.12–2	0.5	1	100		
	Cefuroxime	0.12–4	0.25	0.5	100		
	Trimethoprim-sulphamethoxazole	0.03–32	0.03	1	88.3		
	Tetracycline	0.12–16	0.5	4	88.3		
	Levofloxacin	0.008–8	0.015	0.06	98.4		

^1^Amox/clavulanate = Amoxycillin/clavulanate

^2^No CLSI interpretive criteria for erythromycin and *Haemophilus *spp.

One hundred and three (9.6%) *S. pneumoniae *isolates (from 51 and 53 patients in the 30–64 and >64 year old age groups respectively)) were resistant to both penicillin (MIC ≥ 2 mg/L) and erythromycin (MIC ≥ 1 mg/L) and this was reflected in resistance to amoxycillin, cefuroxime, clarithromycin and azithromycin also (Table 4). These isolates were found in 35 centres in 16 countries. Sixty of these resistant isolates were also resistant to both trimethoprim-sulphamethoxazole and tetracycline. Both telithromycin and levofloxacin had good activity against these isolates, 99% susceptibility to telithromycin and 98.1% to levofloxacin. The MIC_50 _and MIC_90 _values for telithromycin in this population were 0.06 mg/L and 0.5 mg/L, respectively.

**Table 4:** Antibacterial activity against 103 *Streptococcus pneumoniae *isolates with combined macrolide and penicillin resistance

**Antibacterial**	**% susceptible**	**% intermediate**	**% resistant**
Telithromycin	99.0	1.0	0.0
Azithromycin	0	0	100
Clarithromycin	0	0	100
Erythromycin	0	0	100
Penicillin	0	0	100
Amoxycillin	0	0	100
Amoxycillin-clavulanate	72.8	13.6	13.6
Cefuroxime	1.0	1.0	98.0
Trimethoprim-sulphamethoxazole	19.4	16.5	64.1
Tetracycline	11.7	0.0	88.3
Levofloxacin	98.1	0.0	1.9

Over 99% of *H. influenzae *isolates were susceptible to amoxycillin-clavulanate, cefuroxime, telithromycin, azithromycin, and levofloxacin. Tetracycline also had good activity with 97.4% of isolates susceptible. Only 11.7% of *H. influenzae *isolates produced β-lactamase. There were only 60 isolates of *H. parainfluenzae *and 100% of these were susceptible to four of the eight compounds tested, telithromycin, amoxycillin/clavulanate, cefuroxime and azithromycin. Trimethoprim-sulphamethoxazole and tetracycline were the least active compounds. In terms of MICs, levofloxacin, azithromycin and telithromycin were the most potent compounds against *M. catarrhalis *with MIC_90 _values of 0.03 mg/l, 0.06 mg/l and 0.12 mg/l respectively.

There are currently no interpretative NCCLS guidelines available for *M. catarrhalis *to allow classification into susceptible or resistant categories.

The total number of isolates of *S. aureu*s was 335 and of these only 62 were resistant to methicillin (MRSA). Trimethoprim-sulphamethoxazole was the most active compound overall, with 94.9% of all isolates being susceptible. Telithromycin and tetracycline were the next most active with 85.1% and 84.8% of all isolates susceptible. Telithromycin was the most active compound against the MSSA isolates, with 98.9% being susceptible. The susceptibility of MSSA to tetracycline and trimethoprim-sulphamethoxazole was 92.7% and 97.4% susceptible respectively. These three compounds were the only ones to have activity against the MRSA isolates (trimethoprim-sulphamethoxazole 83.9%, tetracycline 50% and telithromycin 24.2%). Less than 10% of the MRSA isolates were susceptible to the remainder of the compounds.

## Discussion

The primary cause of COPD is exposure to tobacco smoke, the major risk factor being cigarette smoking. The demography of the disease in this study and others reflects this, as the majority of patients in this analysis were male and half were elderly (>64 yrs of age) (2). *S. pneumoniae *is most frequently isolated in the least severe cases of AECB, whereas *H. influenzae *is more commonly isolated from moderate to severe cases, with *P. aeruginosa *occurring in severe hospitalised cases [28]. Telithromycin does not have good activity against *Pseudomonas *spp. (GR Micro Limited, data on file, internal report number 141-02-99) and hence may not be an appropriate empirical therapeutic option for AECB patients with severe underlying disease who are hospitalized for an acute exacerbation.

Whether the isolation of a pathogen during AECB represents an infection responsible for the exacerbation has been debated for many years [29-31]. Bacteria have been isolated almost as frequently from patients with stable COPD as those with an AECB, and clinical trials of antibiotic therapy in AECB show contradictory and sometimes unconvincing results [30]. The presence of bacteria in the lower airways is, however, regarded as abnormal since these airways are sterile in healthy adults, and it has been hypothesized that the presence of bacteria in stable COPD represents a low-grade smouldering infection. In addition, a recent study has shown that infection with different strains of pathogens that are new to the patient is associated with development of exacerbation [32,33].

Amoxycillin-clavulanate, azithromycin, and levofloxacin have been shown to be effective in the treatment of AECB, however, there is concern regarding their long-term usefulness, because of the development of resistance to these agents among the causative pathogens [34,35]. Telithromycin has a more focused spectrum of activity than the β-lactams and the fluoroquinolones; it is specifically targeted against pathogens causing community-acquired respiratory disease, including those most commonly associated with AECB. In addition, it is active against penicillin- and macrolide-resistant strains of *S. pneumoniae *and hence offers a viable potential option for the empiric treatment of AECB in non-hospitalised patients [36].

The data in this study demonstrate that telithromycin has high *in vitro *activity against the commonest bacterial pathogens causing AECB. These data also show that telithromycin has the highest overall activity against bacterial isolates from patients with AECB, regardless of species. Almost 10% of *S. pneumoniae *isolated were resistant to penicillin, macrolides, and at least one of the other antibiotics tested, with only telithromycin and levofloxacin retaining high activity against these isolates (99.0% and 98.1%, respectively). The validity of this finding is strengthened as the isolates were obtained from a large number of patients over a wide geographical distribution.

Although atypical pathogens were not examined in the PROTEKT study, telithromycin has been shown to have superior activity *in vitro *against *Chlamydophila pneumoniae *to the other macrolides with the exception of clarithromycin and has similar activity to the fluoroquinolones [37]. In guinea pig models, telithromycin had better activity than erythromycin against *Legionella pneumophila *infections [38]. *In vitro*, the activity of telithromycin against *L. pneumophila *was similar to levofloxacin but better than erythromycin [38]. β-lactams and cephalosporins have no activity against *Mycoplasma pneumoniae *as this species lacks a typical bacterial cell wall, the site of activity for these drugs. Telithromycin has been found to have higher activity than doxycycline and levofloxacin against *M. pneumoniae *[39]. As the atypical pathogens can represent up to 10% of infections associated with AECB, the efficacy of telithromycin against these pathogens could be a consideration in the selection of empiric therapy for AECB.

Telithromycin has been shown to penetrate into respiratory tissues well [40]. The concentration of telithromycin in alveolar macrophages and epithelial lining fluid exceeds that of plasma markedly and remains at therapeutic levels for 24 hours after dosing. Bactericidal levels are also maintained in plasma. A good post-antibiotic effect has also been observed [41]. Telithromycin causes only moderate ecological disturbance to oral and intestinal flora comparable to that associated with clarithromycin and it does not significantly increase the development of resistance in the normal flora, although the MIC of oral streptococci can be slightly raised [42].

Telithromycin can be administered once a day for AECB. Clinical studies have demonstrated that 800 mg administered once daily for 5 days was as effective and well tolerated as a 10-day course of amoxycillin/clavulanate (500/125 mg 3 times daily for 10 days), cefuroxime axetil (500 mg twice daily for 10 days) or clarithromycin (500 mg twice daily for 10 days) [43]. Other clinical studies have also confirmed the safety and tolerability of telithromycin 800 mg administered for 5 – 10 days [44]. Once a day dosing schedules and shorter courses may promote patient adherence to therapy, and this in turn could delay the development of resistance.

Although this study provides valuable information on the overall antimicrobial profile of bacteria causing AECB, care should be taken when interpreting data related to specific demographics. The prevalence of species could not be calculated in this study as a major limitation, inherent to most surveillance studies, is the requirement for collecting centres to fulfil a specified quota of isolates over a defined time period (1 year). If, for instance, a centre managed to fulfil the quota for *S. pneumoniae *isolates from patients with community-acquired pneumonia, it could then only send *H. influenzae *from patients with AECB to fulfil the quota for this organism. In addition, atypical pathogens were not sampled and they can represent up to 10% of the causative pathogens [28].

In summary, the data presented here demonstrate that telithromycin has good *in vitro *activity against *H. influenzae*, *S. pneumoniae*, and *M. catarrhalis*, respiratory pathogens commonly isolated in AECB. It is as active as or more active than agents that are currently used in this clinical setting. Additionally, although not shown here, telithromycin has better *in vitro *activity against atypical pathogens than other agents; an important advantage in this clinical setting as these pathogens may represent 10% of AECB associated infections.

The development of resistance will always be a threat to the usefulness of antibacterial compounds, however surveillance studies such as PROTEKT allow the rapid detection and characterization of resistance mechanisms and highlight the need for and examine the *in vitro *efficacy of newer antibacterial agents. Providing careful surveillance for the development of resistance is maintained, telithromycin currently offers a useful agent in the treatment of AECB.
